# Pathological characteristics of cervical lymph node metastases in papillary thyroid carcinoma compared with lung adenocarcinoma

**DOI:** 10.3389/fonc.2026.1885180

**Published:** 2026-09-04

**Authors:** Mei-Xuan Shao, Yun Niu, Ying Wei, Zhen-Long Zhao, Ming-An Yu

**Affiliations:** 1 Chinese Academy of Medical Sciences & Peking Union Medical College, Beijing, China; 2 Department of Interventional Medicine, China–Japan Friendship Hospital, Beijing, China; 3 Department of Pathology, China–Japan Friendship Hospital, Beijing, China

**Keywords:** biological marker, lung adecarcinoma, lymph node metastasis, proliferation, thyroid carcinoma

## Abstract

**Background:**

US-guided thermal ablation (TA) has shown favorable local control for cervical lymph node metastases (LNM) from papillary thyroid carcinoma (PTC), whereas outcomes appear less favorable for LNM from lung adenocarcinoma (LAC). The pathological basis underlying these differences remains unclear.

**Purpose:**

To compare the pathological characteristics of cervical LNMs from PTC and LAC, focusing on proliferation- and invasion-related markers, and to explore potential biological factors associated with their distinct clinical behavior.

**Methods:**

In this single-center retrospective study, cervical LNM specimens from patients with PTC or LAC were assessed by immunohistochemistry for Ki-67, PCNA, p53, and MMP-9. Marker expression in tumor cells was compared between groups.

**Results:**

A total of 145 patients were included (PTC-N1a, n = 50; PTC-N1b, n = 50; LAC-LNM, n = 45). LAC-related lymph node metastases (LAC-LNM) showed significantly higher Ki-67, PCNA, p53, and MMP-9 staining intensity than both PTC subgroups (all overall P < 0.001). The proportion of MMP-9-positive tumor cells also differed among groups (P = 0.016). Within the PTC cohort, only Ki-67 expression was significantly higher in N1b than in N1a (P = 0.001). After adjustment for age and sex, LAC-LNM remained associated with higher Ki-67, PCNA, and p53 expression and greater MMP-9 staining intensity, whereas the difference in MMP-9-positive tumor cell proportion was no longer significant.

**Conclusion:**

LAC-LNM exhibited a more proliferative immunophenotype and greater MMP-9 staining intensity than PTC-LNM. These differences may contribute to their distinct clinical behavior.

## Introduction

Papillary thyroid carcinoma (PTC) is the most common subtype of thyroid cancer, accounting for 70%-90% of differentiated thyroid cancers (1, 2). It generally follows an indolent clinical course but is frequently associated with regional disease spread, with cervical lymph node metastases (LNM) reported in 30% to 80% of patients (3, 4). In recent years, ultrasound (US)-guided thermal ablation (TA) has emerged as an effective treatment option for selected patients with PTC, and current guidelines recognize percutaneous ablation as an alternative to active surveillance or surgical resection for patients with T1aN0M0 disease (5). Building on this experience, TA has also been applied to PTC patients with LNM, demonstrating favorable outcomes and local tumor control comparable to surgical treatment, while offering the advantages of minimal invasiveness, preservation of thyroid function, and reduced procedure-related morbidity (6).

Beyond PTC, TA has also been applied to LNM from other malignancies, including lung adenocarcinoma (LAC), particularly in patients who are not candidates for surgery or have limited treatment options after systemic therapy (7, 8). However, existing studies have shown that treatment outcomes after TA differ across tumor types. Compared with PTC-related lymph node metastases (PTC-LNM), LAC-related lymph node metastases (LAC-LNM) are associated with higher rates of post-ablation disease progression (9). These findings suggest that LNM originating from different primary tumors may exhibit heterogeneous responses to TA. The underlying biological mechanisms responsible for these differences remain poorly understood. Therefore, the present study aimed to compare the pathological characteristics of LNM from PTC and LAC, with a focus on proliferation- and invasion-related markers, and to explore potential biological factors associated with their distinct clinical behavior.

## Materials and methods

This was a single-center retrospective study conducted at the China-Japan Friendship Hospital. The study protocol was approved by the Human Ethics Review Committee of the China-Japan Friendship Hospital. Written informed consent for the surgical or biopsy procedures was obtained from all patients at the time of clinical care. Owing to the retrospective design and the use of anonymized data, the requirement for additional informed consent for participation in this study was waived.

### Patients

This retrospective study enrolled consecutive patients with LNM from PTC or LAC who underwent surgical lymph node resection or US-guided core-needle biopsy (CNB) between June 2014 and September 2020.

The inclusion criteria for patients were as follows: (1) PTC-LNM confirmed by histopathological examination of surgically resected specimens, or LAC-LNM confirmed by CNB; (2) availability of sufficient clinical and pathological data for semiquantitative immunohistochemical evaluation.

The exclusion criteria were as follows: (1) insufficient or poorly preserved tissue for immunohistochemical analysis; (2) patients with a history of head and neck radiotherapy or other locoregional treatments.

### Histopathological and immunohistochemical analysis

All lymph node specimens were fixed in 10% neutral buffered formalin and embedded in paraffin. Serial 4-µm sections were cut and stained with hematoxylin and eosin (H&E) for routine histopathological evaluation. H&E-stained sections from all cases were independently reviewed by two experienced pathologists who were blinded to the clinical data. The reviewers confirmed the diagnosis of metastatic PTC or LAC, documented the distribution and pattern of metastatic tumor within the cervical lymph nodes, and recorded the involved cervical lymph node levels. Discrepant interpretations were resolved by joint review and consensus.

Immunohistochemical (IHC) staining was performed on representative formalin-fixed, paraffin-embedded sections using an automated immunostainer and a streptavidin-biotin-horseradish peroxidase-diaminobenzidine (DAB) detection system, in accordance with the manufacturer’ s instructions. CNB specimens were considered adequate for immunohistochemical evaluation based on prior validation studies. Monoclonal antibodies against Ki-67, PCNA, p53, and MMP-9 (all purchased from Beijing Zhongshan Golden Bridge Biotechnology Co., Ltd., Beijing, China) were used.

### Evaluation of immunohistochemical staining

IHC-stained slides were independently assessed by the same two pathologists, who were blinded to all clinical information and group allocation. For each marker, areas with the highest density of positive cells were selected at low magnification, and tumor cells and macrophages were quantified at high power (×400). For each case, the final values represented the average of the two observers; in the event of substantial discrepancies, the slides were re-examined together and a consensus value was recorded.

### Tumor cell markers (Ki-67, PCNA, and p53)

Ki-67, PCNA, and p53 were evaluated as nuclear staining in metastatic tumor cells. For each marker, the labeling index was defined as the percentage of positively stained tumor nuclei among the total number of tumor cells counted, expressed as an integer from 0% to 100%. These values were treated as continuous variables for statistical analysis.

### MMP-9 in tumor cells

MMP-9 expression in metastatic tumor cells was defined as cytoplasmic and/or membranous DAB staining in tumor cells. Two parameters were assessed: (1) the proportion of positive tumor cells, defined as the percentage of MMP-9-positive tumor cells among all tumor cells in the evaluated high-power fields (0-100%); and (2) the staining intensity in tumor cells, scored on a four-tier scale as 0 (no staining), 1+ (weak), 2+ (moderate), or 3+ (strong).

Based on pathological and imaging findings, patients were first classified into two primary cohorts: the PTC-LNM cohort and the LAC-LNM cohort. According to the American Joint Committee on Cancer (AJCC) nodal classification, the PTC cohort was further subdivided into two nodal subgroups for subsequent three-group comparisons: PTC-N1a, defined as PTC with central compartment (levels VI/VII) LNM, and PTC-N1b, defined as PTC with lateral neck (levels II-V) LNM with or without concomitant central compartment (levels VI/VII) involvement. The LAC-LNM group comprised patients with primary LAC and LNM confirmed by CNB.

### Statistical analysis

Statistical analyses were performed using SPSS software (version 26.0; IBM Corp., Armonk, NY, USA) and R software (version 4.3.2; R Foundation for Statistical Computing, Vienna, Austria). Continuous data are presented as means ± standard deviations when approximately normally distributed and as medians with interquartile ranges (IQRs) when non-normally distributed. Categorical variables are presented as numbers and percentages.

Overall comparisons of continuous variables among the PTC-N1a, PTC-N1b, and LAC-LNM groups were performed using the Kruskal-Wallis test. When an overall difference was identified, pairwise comparisons were conducted using the Mann-Whitney U test with Bonferroni adjustment for multiple comparisons. Because Bonferroni-adjusted P values are reported, an adjusted P value < 0.05 was considered statistically significant.

For clinicopathological characteristics available only in the PTC cohort, continuous variables were compared between PTC-N1a and PTC-N1b using the Mann-Whitney U test. Categorical variables were compared using the chi-square test or Fisher’ s exact test, as appropriate.

To determine whether differences in immunohistochemical marker expression between LAC-LNM and PTC-LNM persisted after adjustment for age and sex, the PTC-N1a and PTC-N1b groups were combined as PTC-LNM. Ki-67, PCNA, p53, and the proportion of MMP-9-positive tumor cells were analyzed using median regression with bootstrap inference. Effect estimates represent the adjusted median difference in marker expression for LAC-LNM relative to PTC-LNM.

MMP-9 staining intensity was treated as an ordinal outcome and analyzed using ordinal logistic regression. Results are presented as common odds ratios (ORs) with 95% confidence intervals (CIs), with an OR > 1 indicating greater odds of being in a higher staining-intensity category in LAC-LNM than in PTC-LNM. The proportional odds assumption was assessed, and all multivariable models were adjusted for age and sex.

All statistical tests were two-sided. Except for the reported Bonferroni-adjusted pairwise comparisons, a P value < 0.05 was considered statistically significant.

## Results

### Baseline demographic and clinicopathological characteristics

A total of 145 patients were included in this study, comprising 50 patients with PTC-N1a, 50 with PTC-N1b, and 45 with LAC-LNM. The median age was 43.0 years (IQR, 38.0-53.8) in the PTC-N1a group, 34.0 years (IQR, 31.0-49.2) in the PTC-N1b group, and 66.0 years (IQR, 55.0-71.0) in the LAC-LNM group, with a significant overall difference among the three groups (P < 0.001). Male patients accounted for 38.0%, 44.0%, and 62.2% of the respective groups; however, the overall difference in sex distribution did not reach statistical significance (P = 0.051) (**Table 1**).

**Table 1:** Baseline demographic and clinicopathological characteristics of the study population.

Characteristic	PTC-N1a (n = 50)	PTC-N1b (n = 50)	LAC-LNM (n = 45)	P value
Age, years	43.0 (38.0-53.8)	34.0 (31.0-49.2)	66.0 (55.0-71.0)	<0.001^a^
Male sex, n (%)	19 (38.0%)	22 (44.0%)	28 (62.2%)	0.051^a^
Primary tumor maximum diameter, cm	0.80 (0.60-1.20)	1.00 (0.72-1.58)	—	0.083^b^
Number of tumor foci	1 (1–2)	1 (1–2)	—	0.343^b^
Number of LNM	3 (2–5)	9 (5–14)	—	<0.001^b^
Hashimoto thyroiditis, n (%)	12 (24.0%)	14 (28.0%)	—	0.648^b^

Data are presented as median (IQR) or n (%).

^a^ Overall comparison among PTC-N1a, PTC-N1b, and LAC-LNM.

^b^ Comparison between PTC-N1a and PTC-N1b.

PTC, papillary thyroid carcinoma; N1a, central compartment lymph node metastasis; N1b, lateral cervical lymph node metastasis; LAC, lung adenocarcinoma; LNM, lymph node metastasis; IQR, interquartile range.

Within the PTC cohort, the median maximum primary tumor diameter was 0.80 cm (IQR, 0.60-1.20) in PTC-N1a and 1.00 cm (IQR, 0.72-1.58) in PTC-N1b, with no statistically significant difference between the two subgroups (P = 0.083). The median number of tumor foci was 1 (IQR, 1-2) in both groups (P = 0.343). In contrast, the number of LNM was significantly greater in PTC-N1b than in PTC-N1a, with median values of 9 (IQR, 5-14) and 3 (IQR, 2-5), respectively (P < 0.001). Hashimoto thyroiditis was present in 24.0% of patients with PTC-N1a and 28.0% of those with PTC-N1b, with no significant difference between the subgroups (P = 0.648).

### Comparison of immunohistochemical marker expression among PTC-N1a, PTC-N1b, and LAC-LNM

**Figure 1:**
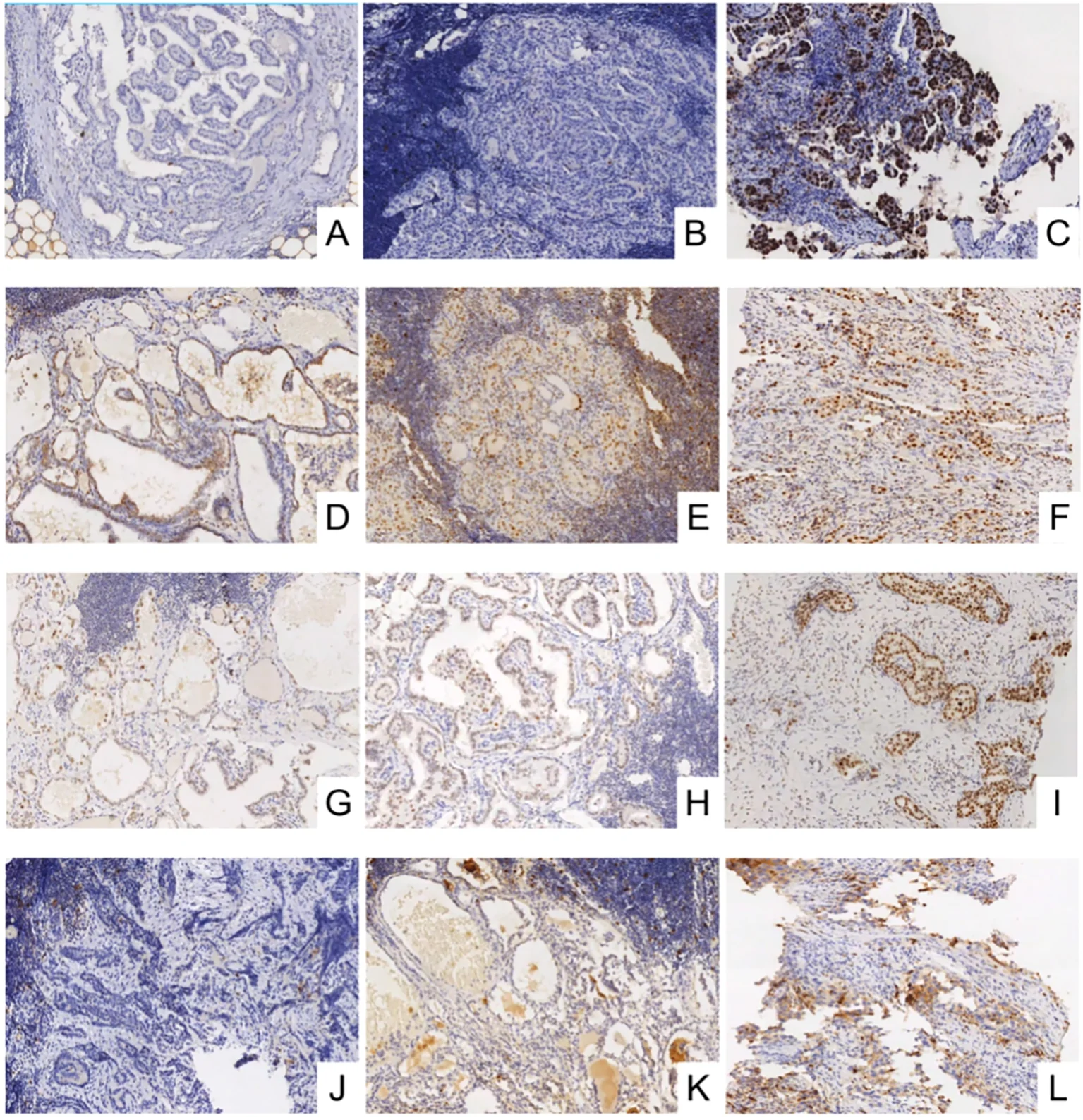
Representative immunohistochemical staining of proliferation and invasion markers in PTC-N1a, PTC-N1b, and LAC-LNM tissues. Ki-67 **(A–C)**, PCNA **(D–F)**, p53 **(G–I)**, and MMP-9 **(J–L)** are shown, with images arranged from left to right as PTC-N1a, PTC-N1b, and LAC-LNM. Original magnification ×200; scale bar = 50 μm.

Marked immunohistochemical differences were observed among PTC-N1a, PTC-N1b, and LAC-LNM (**Figure 1**) (**Table 2**). Ki-67 expression differed significantly among the three groups (overall P < 0.001). The median Ki-67 index was 40.0% (IQR, 25.0-50.0) in LAC-LNM, compared with 2.0% (IQR, 1.0-2.0) in PTC-N1a and 2.0% (IQR, 2.0-5.0) in PTC-N1b. Bonferroni-adjusted pairwise comparisons showed that Ki-67 expression was significantly higher in LAC-LNM than in either PTC subgroup (both P < 0.001). Within the PTC cohort, Ki-67 expression was also significantly higher in PTC-N1b than in PTC-N1a (P = 0.001).

**Table 2:** Comparison of immunohistochemical marker expression among PTC-N1a, PTC-N1b, and LAC-LNM.

Marker	Marker expression, median (IQR)			Overall P value	Pairwise P value		
	PTC-N1a (n = 50)	PTC-N1b (n = 50)	LAC-LNM (n = 45)		N1a vs N1b	N1a vs LAC	N1b vs LAC
Ki-67, %	2.0 (1.0-2.0)	2.0 (2.0-5.0)	40.0 (25.0-50.0)	<0.001	0.001	<0.001	<0.001
PCNA, %	9.0 (2.0-10.0)	10.0 (3.5-20.0)	50.0 (30.0-80.0)	<0.001	0.237	<0.001	<0.001
p53, %	3.0 (2.0-5.0)	2.0 (1.0-3.0)	50.0 (2.0-90.0)	<0.001	0.078	<0.001	<0.001
MMP-9-positive tumor cells, %	1.0 (1.0-2.0)	1.0 (1.0-2.0)	2.0 (1.0-10.0)	0.016	1.000	0.036	0.055
MMP-9 staining intensity, +	1 (1–1)	1 (1–1)	2 (1–2)	<0.001	1.000	<0.001	<0.001

Overall P values were obtained using the Kruskal–Wallis test. Pairwise comparisons were performed using the Mann–Whitney U test with Bonferroni correction. PTC, papillary thyroid carcinoma; N1a, central compartment lymph node metastasis; N1b, lateral cervical lymph node metastasis; LAC, lung adenocarcinoma; LNM, lymph node metastasis; IQR, interquartile range.

PCNA expression also showed a significant overall difference among the three groups (P < 0.001). The median PCNA index was 50.0% (IQR, 30.0-80.0) in LAC-LNM, 9.0% (IQR, 2.0-10.0) in PTC-N1a, and 10.0% (IQR, 3.5-20.0) in PTC-N1b. PCNA expression was significantly higher in LAC-LNM than in both PTC-N1a and PTC-N1b (both adjusted P < 0.001), whereas no significant difference was observed between the two PTC subgroups (adjusted P = 0.237).

For p53, the median expression level was 50.0% (IQR, 2.0-90.0) in LAC-LNM, compared with 3.0% (IQR, 2.0-5.0) in PTC-N1a and 2.0% (IQR, 1.0-3.0) in PTC-N1b. The overall difference was statistically significant (P < 0.001), and LAC-LNM showed significantly higher p53 expression than either PTC subgroup (both adjusted P < 0.001). No significant difference was detected between PTC-N1a and PTC-N1b after correction for multiple comparisons (adjusted P = 0.078).

The proportion of MMP-9-positive tumor cells differed significantly among the three groups (overall P = 0.016). In Bonferroni-adjusted pairwise comparisons, the proportion was higher in LAC-LNM than in PTC-N1a (adjusted P = 0.036), whereas the difference between LAC-LNM and PTC-N1b did not reach statistical significance (adjusted P = 0.055). No difference was observed between PTC-N1a and PTC-N1b (adjusted P = 1.000).

MMP-9 staining intensity differed significantly among the three groups (overall P < 0.001). The median staining intensity was 2(IQR, 1-2) in LAC-LNM and 1(IQR, 1-1) in both PTC subgroups. Staining intensity was significantly greater in LAC-LNM than in PTC-N1a and PTC-N1b (both adjusted P < 0.001), whereas no significant difference was found between the two PTC subgroups (adjusted P = 1.000).

Overall, LAC-LNM exhibited a distinct immunohistochemical profile characterized by higher Ki-67 and PCNA expression, higher p53 expression, and greater MMP-9 staining intensity. In contrast, the difference in the proportion of MMP-9-positive tumor cells was relatively modest and did not remain statistically significant in the adjusted pairwise comparisons.

### Age- and sex-adjusted differences between LAC-LNM and PTC-LNM

To determine whether the observed immunohistochemical differences persisted after accounting for the demographic imbalance between the groups, PTC-N1a and PTC-N1b were combined as PTC-LNM, and age- and sex-adjusted analyses were performed (**Table 3**).

**Table 3:** Age- and sex-adjusted differences in immunohistochemical marker expression between LAC-LNM and PTC-LNM.

Marker	Model	Adjusted effect (95% CI)	P value
Ki-67, %	Median regression	39.04 (32.46-45.61)	<0.001
PCNA, %	Median regression	40.00 (20.98-59.02)	<0.001
p53, %	Median regression	48.00 (12.54-83.46)	0.009
MMP-9-positive tumor cells, %	Median regression	1.00 (-0.62-2.62)	0.228
MMP-9 staining intensity, +	Ordinal logistic regression	26.16 (7.42-92.25)	<0.001

PTC-N1a and PTC-N1b were combined as PTC-LNM. CI, confidence interval.

In median regression analyses, LAC-LNM remained associated with substantially higher Ki-67 expression after adjustment for age and sex. The adjusted median Ki-67 level was estimated to be 39.04 percentage points higher in LAC-LNM than in PTC-LNM (95% CI, 32.46-45.61; P < 0.001). Similarly, the adjusted median PCNA level was 40.00 percentage points higher in LAC-LNM (95% CI, 20.98-59.02; P < 0.001), and the adjusted median p53 level was 48.00 percentage points higher (95% CI, 12.54-83.46; P = 0.009).

By contrast, the adjusted difference in the proportion of MMP-9-positive tumor cells was not statistically significant. LAC-LNM showed an estimated median increase of 1.00 percentage point relative to PTC-LNM, but the confidence interval included zero (95% CI, −0.62 to 2.62; P = 0.228).

In the ordinal logistic regression analysis, LAC-LNM was strongly associated with higher MMP-9 staining intensity after adjustment for age and sex. The common odds of being in a higher staining category were 26.16 times greater in LAC-LNM than in PTC-LNM (95% CI, 7.42-92.25; P < 0.001).

Overall, the higher expression levels of Ki-67, PCNA, and p53 and the greater MMP-9 staining intensity observed in LAC-LNM could not be explained solely by differences in age or sex. In contrast, the proportion of MMP-9-positive tumor cells was not independently different between LAC-LNM and PTC-LNM after adjustment for these covariates.

## Discussion

LNM from different primary tumors may exhibit distinct biological characteristics that contribute to heterogeneous clinical behavior and treatment response. Previous observations suggested that LAC-LNM may be more prone to progression after ablation than PTC-LNM, but the underlying biological basis remains unclear. Therefore, the present study compared the immunohistochemical profiles of LAC-LNM and PTC-LNM, focusing on biological differences.

The selected biomarkers were chosen to capture key biological features related to tumor growth, invasion, and LNM in PTC and LAC. Ki-67 and PCNA were included as proliferation-related markers. Ki-67 is expressed during the active phases of the cell cycle but is absent in quiescent cells. In PTC, a higher Ki-67 labeling index has been associated with LNM, greater metastatic nodal burden, higher N stage, and shorter disease-free survival (10). In LAC, increased Ki-67 expression has been associated with LNM, high-grade histologic patterns, poor differentiation, and unfavorable survival (11, 12). In addition to Ki-67, PCNA provides complementary information on tumor-cell proliferation through its involvement in DNA replication and repair. Previous studies in both LAC and PTC have shown that PCNA expression reflects tumor-cell proliferative activity and may be associated with aggressive clinicopathologic features, although its prognostic significance has not been consistent across studies (13–15). p53 was selected to reflect dysregulation of a major tumor-suppressor pathway involved in DNA-damage response, cell-cycle arrest, senescence, and apoptosis. In PTC, p53 overexpression has been associated with larger tumor size, LNM, and greater metastatic nodal burden (16). In non-small cell lung cancer, particularly LAC, TP53 alterations have been associated with aggressive tumor biology and adverse clinical outcomes (17, 18). MMP-9 was selected as a marker of extracellular-matrix remodeling and invasive potential because it contributes to basement-membrane degradation and may facilitate tumor-cell migration, stromal invasion, and metastatic dissemination. In PTC, increased MMP-9 expression has been associated with LNM, extrathyroidal invasion, greater tumor infiltration, and advanced tumor stage (19, 20). In LAC, MMP-9 overexpression has been associated with lymph node status, invasive behavior, and metastasis-related phenotypes (21). Taken together, these four markers were intended to provide a multidimensional assessment of proliferative activity and extracellular-matrix remodeling capacity in LNM from different primary tumors.

Marked differences in immunohistochemical profiles were observed between LAC-LNM and PTC-LNM. LAC-LNM showed substantially higher expression of the proliferation markers Ki-67 and PCNA, as well as higher p53 expression, than either PTC subgroup. Importantly, these differences remained significant after adjustment for the marked age and sex imbalance between LAC-LNM and PTC-LNM. Median regression showed that, compared with PTC-LNM, LAC-LNM had adjusted increases of 39.04 percentage points in Ki-67, 40.00 percentage points in PCNA, and 48.00 percentage points in p53 expression.

The markedly elevated Ki-67 and PCNA expression observed in LAC-LNM suggests a substantially higher proliferative capacity than that of PTC-LNM. This biological profile is consistent with the more aggressive clinical course generally associated with metastatic LAC. However, because the present study did not directly examine tumor growth rates, treatment response, or survival, a causal relationship between marker expression and clinical progression cannot be established. Rather, the findings provide a potential biological explanation for previously reported differences in the behavior of LNM from different primary tumors.

Within the PTC cohort, Ki-67 expression was modestly but significantly higher in PTC-N1b than in PTC-N1a. In contrast, PCNA and p53 expression did not differ significantly between the two PTC subgroups after correction for multiple comparisons. The higher Ki-67 expression in PTC-N1b may reflect an association between greater proliferative activity and lateral LNM. Nevertheless, the absolute Ki-67 levels in both PTC subgroups remained substantially lower than those in LAC-LNM. Thus, even in the presence of more extensive nodal disease, PTC-LNM retained a comparatively low-proliferative immunophenotype. This contrast may partly account for the generally more indolent clinical behavior of PTC-LNM compared with LAC-LNM.

MMP-9 staining intensity was consistently greater in LAC-LNM than in both PTC subgroups. This difference remained pronounced after adjustment for age and sex, with LAC-LNM showing substantially greater odds of being classified into a higher staining-intensity category. In contrast, the proportion of MMP-9-positive tumor cells did not differ significantly between LAC-LNM and PTC-LNM after adjustment. These findings suggest that the level of MMP-9 expression within positive tumor cells, rather than the proportion of positive cells alone, may more clearly distinguish LAC-LNM from PTC-LNM. Greater MMP-9 staining intensity may reflect enhanced extracellular-matrix remodeling and invasive potential, which could theoretically promote microscopic tumor extension beyond imaging-defined lesion margins and thereby increase the difficulty of achieving complete local treatment. However, because microscopic extension and treatment outcomes were not directly assessed in this study, this potential association requires further investigation.

Collectively, these findings demonstrate distinct immunohistochemical profiles between LAC-LNM and PTC-LNM. The higher expression of Ki-67 and PCNA, together with greater MMP-9 staining intensity in LAC-LNM, may be relevant to differences in biological behavior and local treatment response. However, because treatment outcomes were not assessed in the present study, it remains unclear whether these markers are associated with local progression, recurrence, or treatment efficacy. Prospective studies integrating immunohistochemical findings with ablation parameters and longitudinal outcomes are needed to determine their potential value in treatment selection and clinical risk stratification.

Several limitations should be acknowledged. First, this was a retrospective study, and selection bias and unmeasured confounding cannot be excluded. Although the adjusted analyses accounted for age and sex, residual confounding by other clinical, anatomical, or molecular characteristics may remain. Second, the study focused on a limited panel of immunohistochemical markers and did not include direct genomic analysis. In particular, p53 immunohistochemical expression should not be considered equivalent to TP53 mutation status. Third, the study did not assess immune-related markers or the tumor microenvironment, and future studies incorporating these factors are warranted.

In addition, sampling method was completely confounded with tumor type because all LAC-LNM specimens were obtained by CNB, whereas all PTC-LNM specimens were obtained by surgical resection. Therefore, the observed differences could not be fully separated from potential effects related to tissue volume, architectural preservation, and intratumoral sampling heterogeneity. Although CNB generally provides sufficient material for routine immunohistochemical assessment, it may not fully capture intratumoral heterogeneity. Finally, the sample size was relatively limited, and the study was conducted within a single clinical setting. External validation in larger, multicenter cohorts is required to assess the generalizability of these findings.

## Conclusion

In this study, LAC-LNM exhibited substantially higher Ki-67, PCNA, and p53 expression and greater MMP-9 staining intensity than PTC-LNM, and these differences persisted after adjustment for age and sex. In contrast, the proportion of MMP-9-positive tumor cells did not independently differ between the two groups. Within the PTC cohort, Ki-67 expression was higher in PTC-N1b than in PTC-N1a, whereas PCNA, p53, and MMP-9 expression showed no significant subgroup differences after correction for multiple comparisons. These findings demonstrate distinct immunohistochemical profiles between LAC-LNM and PTC-LNM and may provide a biological explanation for their heterogeneous clinical behavior. Further prospective studies incorporating treatment outcomes are needed to determine whether these markers can improve risk stratification and individualized management.

## Data Availability

The raw data supporting the conclusions of this article will be made available by the authors, without undue reservation.
